# Discrimination and authentication of geographical origin of Turkish Taşköprü garlic by investigating volatile organosulfur compound profiles and multivariate analyses

**DOI:** 10.55730/1300-0527.3423

**Published:** 2022-04-05

**Authors:** Orhan DESTANOĞLU

**Affiliations:** Department of Science, Institute of Forensic Sciences and Legal Medicine, İstanbul University-Cerrahpaşa, İstanbul, Turkey

**Keywords:** Geographical indication, garlic, organosulfur compounds, traceability, chemometrics

## Abstract

Nowadays, counterfeiting and adulteration on foods take place around the world in a variety of ways. Identification and authentication of geographical origin of agricultural products has great importance not only for food safety, but for protection of registrations as well. This study aimed at discriminating the Turkish Taşköprü garlic, possessing protected geographical indication (GI) in Turkey and GI registration from the European Union, from the other samples. For this reason, the combination of headspace-gas chromatography-mass spectrometry (HS-GC-MS) analysis of the volatile organosulfur compounds (VOSCs) and two multivariate analysis techniques, namely hierarchical cluster analysis (HCA) and principal component analysis (PCA), were employed for classifying the garlic samples on the basis of their geographical origin. Discrimination of Taşköprü garlic and the other samples including a suspicious sample imported from China was accomplished by performing two dimensional and three dimensional PCA analyses to relative amounts of VOSCs and also to chromatogram raw data.

## 1. Introduction

Garlic (*Allium sativum* L.) is a plant of the genus *Allium* which belongs to the family Alliaceae. Genus *Allium* consists of onions, leeks, chives, and more than 800 other members. Among the members of the genus *Allium*, only a few have been cultivated as foods [1]. Garlic has been cultivated since ancient times and is still used for culinary and medicinal purposes due to its health benefits. Its unique pungent aroma and flavor are derived from the organosulfur compounds [2]. In addition to organosulfur compounds, it also possesses proteins, carbohydrates, oils, minerals (phosphorus, potassium, calcium, and magnesium), vitamins, free amino acids, and alcohols [2–6]. Thus, it is more nutritious than the other bulb crops [7]. Some clinical studies have investigated the garlic’s therapeutic properties on the diseases such as yeast and bacterial infections, hypertension, cancer, hypercholesterolemia, and inflammation [4,8].

When looking at garlic production in the world, according to FAO data, 1.13 billion tons of vegetables were produced on 59.7 million hectares of land in the world in 2019, of which 2.7% is garlic. The world’s top three (major) garlic cultivation areas are in China, India, and Bangladesh. 20.7 million tons of garlic was produced in China while 2.9 million tons of garlic was produced in India in 2019.China, which is the world leader in garlic production, comprised 75.7% of its total production [9]. In 2019, dry garlic cultivation area in Turkey increased by 5.7% compared to 2018 and reached 12 thousand hectares.

According to Commission Implementing Regulation (EU) 2021/615 released on 16 April 2021, designation of origin of the Taşköprü garlic, also known as white gold of Taşköprü, was protected and it received geographical indication (GI) registration from the European Union on 7 April 2021 with the specific name of “Taşköprü Sarımsağı” [10]. Kastamonu is the largest province on account of dry garlic cultivation area in Turkey with 26.6 thousand decares (21.3%). Taşköprü, an administrative district, is one of the 19 districts of Kastamonu Province in the Black Sea Region of Turkey. The registration document defines the geographical area by “The geographical area covers 1811.31 km^2^. All processes from the planting to the harvesting of “Taşköprü Sarımsağı” must be carried out in Taşköprü district” [11].

Especially today, there are many dimensions of counterfeiting and adulteration on foods. Even though consumers shop more carefully today, those who want to make unfair profits continue to deceive over consumer perception. On the consumer side, one of the most commonly used brands by those who turn the brand region/product relationship into an opportunity in Turkey is Taşköprü garlic. Another city that stands out in garlic production is Gaziantep with well-known “Araban garlic” which is also registered by the Turkish Patent and Trademark Office with geographical indication. However, Araban garlic can be sold under the name Taşköprü in the open market. The reason why some consumers prefer imported garlic from China is its lower price. However, some vendors benefiting from the high course of the market sell Chinese garlic at a price at the same level as Taşköprü garlic. Regardless of its price, Taşköprü garlic is generally preferred in Turkey owing to its taste and strong aroma. It is seen also in the Turkish market that some of the garlic samples with the inscription of domestic production on the label are actually imported from abroad, especially from China. Therefore, authentication of regional and national origin of garlic samples must be distinguished, because the consumers are concerned about the originality of garlic. Thus, sustainability of the quality of the product can be ensured and economic fraud can be prevented by protecting the GI registration.

In the literature, very recent studies on discrimination of *Allium* species and garlic samples originating from different geographical areas across the globe have attempted to examine its elemental profiles, physicochemical properties, isotopic profiles, metabolomics, volatile profiles performing various analytical techniques and statistical methods. Fourteen selected studies from the literature are summarized with the employed target analyses and instruments, and statistical methods are listed in Table 1.

Two of the studies presented in Table 1 discriminated the garlic samples from different regions by investigating the analyses of volatile compounds combined with chemometric methods [13,14]. Mi et al. classified the garlic samples collected from the four major production regions of China by applying PCA and PLS-DA to 55 elements, 68 volatiles (15 alkanes, 9 aldehydes, 3 alcohols, 3 acids, 3 ketones, 2 esters, and 33 other volatile compounds), and 854 metabolites which were previously quantified by ICP-MS, HS-SPME-GC-MS, and UHPLC-Q-TOF/MS, respectively. According to the statistical results, 10 chemical elements, 6 volatiles, and 225 metabolites were suggested as candidate markers for the discrimination of the geographical origins of the garlic samples collected from different regions [13]. In another study, Biancolillo et al. presented a method of combination of HS-SPME/GC-MS analysis of volatile organosulfur compounds (VOSCs) and PLS-DA for classification of red garlic (*Allium sativum* L.) samples grown in four different regions in Italy. Relative (%) peak areas of the identified 13 of VOSCs were used in the chemometric analysis [14].

On the other hand, to discriminate the geographical origins of the garlic samples, NMR and FTIR spectral data have been statistically analyzed with various chemometric tools. Jo et al. achieved to classify the garlic samples and onion samples obtained in Korea and China after analyzing NMR spectral data by PCA [15]. Ritota et al. successfully discriminated red and white garlic samples, which were collected from four different areas in Italy, by examining the NMR data analysis with PLS-DA [16]. Biancolillo et al. presented a nondestructive approach based on ATR-FTIR spectroscopy combined with chemometrics (PLS-DA, SO-CovSel-LDA, and SO-PLS-LDA) to distinguish the garlic samples cultivated in four different regions in Italy [17].

In this study, to statistically elucidate similarities and dissimilarities among the VOSC profiles and the chromatogram raw data, which consisted of thousands of values, obtained from HS-GC-MS analyses, HCA and PCA methods were utilized. Relative contents (%) of the identified 17 of VOSCs were analyzed by HCA and PCA while the chromatograms’ raw data were processed with a PCA application designed for spectral data analyses. The novelty of this article that the method used for PCA for spectroscopy was successfully adapted to chromatograms of the garlic samples cultivated in different regions. Besides, the proposed study did not need further examinations of the other substances such as the other volatiles by using SPME, multielemental profiling, and isotopic ratio analyses for discrimination of the target geographical origins. Moreover, the absence of requirement of additional extraction processes provided these advantages: I) it reduced the sample preparation time and costs, II) the toxic reagents and solvents were neither used during sample preparation nor released to the environment.

Consequently, discriminations of not only Taşköprü garlic and the garlic samples collected from different provinces of Turkey, but also a suspicious sample with the inscription of domestic production and unknown samples, which is important for traceability issue, were achieved by performing the chemometric approach for the first time in the literature.

## 2. Materials and methods

### 2.1. Instrumentation

Separation and determination of VOSCs in garlic samples was carried out by using a PerkinElmer (PE) Clarus 500 GC-MS equipped with a Turbomatrix HS40 headspace autosampler. A free fatty acid phase (FFAP) GC column (PE) with dimensions 30 m length, 0.25 mm i.d., and 0.5 μm df. was utilized for chromatographic separations of VOSCs. Helium was used as the carrier gas at 1 mL/min constant flow rate throughout HS-GC-MS system with HS pressure of 30 psi. All HS-GC-MS conditions are summarized in Table 2. To manage all the parameters of GC-MS system and data acquisition by computer, a TurboMass software was used. Compounds were identified by mass spectral matching in the NIST library.

### 2.2. Samples

The garlic samples from Kahramanmaraş Afşin Koçovası (KMR), Gaziantep Araban (GTP), which have also GI registrations, and Hatay (HTY) were obtained from the different markets in İstanbul while six different Kastamonu Taşköprü (KT) garlic samples were supplied from Taşköprü district of Kastamonu Province. In addition, two samples with no labels and a sample, of which the seller confessed that it was Chinese garlic, with the inscription of domestic production on the label were purchased from local markets and they were coded as suspected samples (SS).

### 2.3. Sample preparation

For each sample, randomly selected garlic clove samples were peeled and then 10-g samples were homogenized by grinding. After 1 g of the homogeneous sample was directly weighed into a 20 mL-HS vial, its lid was tightly closed with the gas-tight teflon silicone cap prior to loading to the HS autosampler.

### 2.4. Data analysis

The statistical analyses were conducted by utilizing the PCA and HCA applications installed in OriginPro software (OriginLab, version 9.6.5.169) after downloading the applications from the official website of the company [26–28]. The reason why PCA was used is that PCA is one of the well-established techniques for dimensionality reducing and interpreting large multivariate data sets with underlying linear structures. PCA is a multivariate analysis technique and its purpose is to extract fundamental or important information from input data into a set of new orthogonal variables called principal components [29]. The PC1 is the most variability of the data, and the PC2 is the next most variability, and other components continue in this order. It, therefore, allows discovering previously unsuspected relationships among the samples.

In this study, PCA was applied in two ways. The relative contents of the VOSCs were processed with PCA application while the raw data of the chromatograms were processed using an easy-to-use application of OriginPro software, namely, PCA for spectroscopy. Even though the latter application was developed for spectra (IR, Fluorescence, UV-Vis, Raman, etc.), successfully discriminative results were also achieved from the chromatograms. On the other hand, the application, namely, heat map with dendrogram (HMD) was used to perform HCA along columns and rows of the relative contents (%) of the VOSCs and samples, respectively, to plot the two-way HMD.

## 3. Results

### 3.1. HS-GC-MS analysis

**Figure 1:**
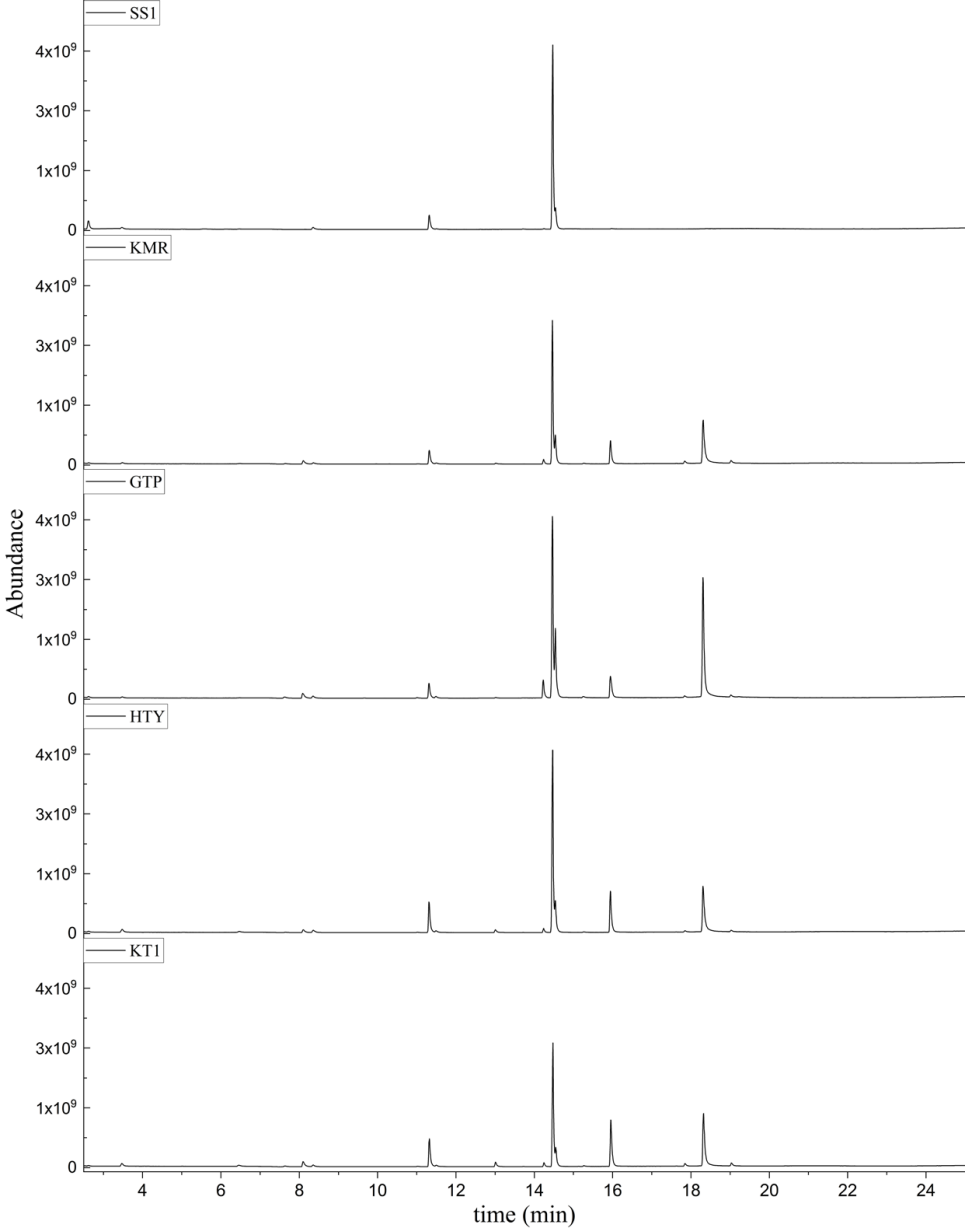
Chromatograms of KT, HTY, GTP, KMR, and SS garlic samples. The retention times (rt) of the analytes are; allyl mercaptan: 2.62 min, allyl methyl sulfide: 3.47 min, dimethyl disulfide: 6.45, diallylsulfide: 8.35, methyl 1-propenyl disulfide: 11.04, methyl 2-propenyl disulfide: 11.32, 1-methyl-2-(prop-1-en-1-yl)disulfane: 11.52, dimethyl trisulfide: 13.02, 1-allyl-2-isopropyldisulfane: 13.71, diallyl disulfide: 14.45, 1-allyl-2-(prop-1-en-1-yl)disulfane: 14.54, 3H-1,2-dithiole: 15.27, methyl 2-propenyl trisulfide: 15.95, 1-allyl-3-propyltrisulfane: 17.62, 3-vinyl-1,2-dithiacyclohex-4-ene: 17.84, di-2-propenyl trisulfide: 18.31, and 2-vinyl-4H-1,3-dithiine: 19.04.

As can be seen in Table 3, the chromatographic profiling of the garlic samples were implemented by investigating 17 VOSCs, which were found as the major compounds under the optimized HS-GC-MS conditions. The chromatograms of the garlic samples are exhibited in Figure 1. In the chromatograms obtained as a result of the analyses performed under the same chromatographic conditions, a noticeable difference was observed as the fewer and smaller peaks on the chromatogram of SS1 sample. The other compounds such as terpenes, aldehydes, alcohols, and the other minor compounds were not detected by using the static HS-GC-MS. The relative contents (%) and molecular structures of the VOSCs examined in this study are demonstrated in Table 3. Among the VOSCs, the most abundant three compounds were diallyl disulfide, di-2-propenyl trisulfide, and methyl 2-propenyl trisulfide in the Turkish garlic samples. Whereas the relative amount of diallyl disulfide was found 63.44% in SS1 which was approximately two-times higher than the average values of the other samples, di-2-propenyl trisulfide and methyl 2-propenyl trisulfide were found 0% and 0.5%, respectively. The highest content value of di-2-propenyl trisulfide was observed in GTP sample with 42.10%. Naturally, it is almost impossible to come to a conclusion by examining numerous data and filtering them. In addition, if an official expert report is to be prepared, the results based on objective values should be presented. Therefore, the present study aimed at utilizing the statistical analyses.

### 3.2. Statistical analysis

#### 3.2.1. HCA and PCA for relative contents of the samples

To statistically determine the differences in 204 data (17 × 12 matrix) given in Table 3, HCA and PCA, which are the unsupervised methods and do not require any labeled or reference data unlike supervised methods, were performed in the present study.

**Figure 2:**
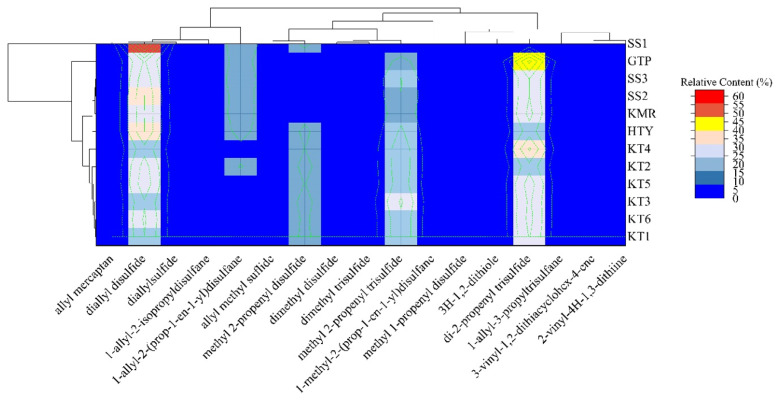
Heat map with dendrogram of the HCA performed on the relative content (%) of the VOSCs in the samples.

Clusterization by HCA provided more apparent results for easily comprehending the differentiations of the garlic samples compared to Table 3. As seen in two-way HMD presented in Figure 2, the horizontal and vertical clusters belong to garlic samples and VOSCs, respectively. It is clear from the clustergram that the first cluster was of the merely SS1 while the second cluster was consisted of subclusters of the Turkish garlic samples from different regions. Since the SS2 and SS3, whose origin were unknown, were found between GTP and KMR, it was concluded that these suspected samples had been grown in Turkey. On the other hand, the third subcluster comprised the only six KT samples.

**Figure 3:**
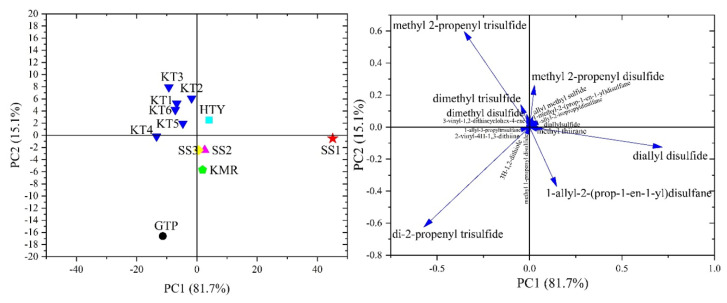
Score plot (left) and loading plot (right) of the PCA for garlic samples by processing relative amounts of VOSCs.

Figure 3 exhibits the resulting score plot and the corresponding loadings of the first two PCs with a total covariance of 96.8%. The corresponding loading plots were used to identify the compounds that allowed the clustering in PCA. Figures 2 and 3 illustrate that in the first cluster of HMD and the vectors in the loading plot for PCA of the relative contents (%), the dominant compounds that distinguish the SS1, GTP, and KT sample from other samples was diallyl disulfide, di-2-propenyl trisulfide, and methyl 2-propenyl trisulfide, respectively. Also, methyl 2-propenyl disulfide was the compound to differentiate SS1 from the closest sample GTP. All samples contained diallyl disulfide with different percentages though only SS1 did not possess di-2-propenyl trisulfide and methyl 2-propenyl trisulfide, which was another decisive difference. It was observed from PCA of relative contents of the samples, KT, GTP, HTY, and KMR samples were distributed in the top-left, bottom-left, top-right, and bottom-right quadrants, respectively.

As in the 3rd subcluster of HCA, KT samples were accumulated in a separate quadrant as a result of PCA applied to the relative contents. Moreover, KMR was seen as the closer sample for SS2 and SS3. On the other hand, according to Figures 2 and 3, KT was distinctly separated from the other samples grown different regions in Turkey with these compounds; diallyl disulfide for HTY, di-2-propenyl trisulfide (major one) and 1-allyl-2-(prop-1-en-1-yl)disulfane for GTP, and 1-allyl-2-(prop-1-en-1-yl)disulfane for KMR.

#### 3.2.2. PCA for chromatograms

The raw data of the chromatograms exported from the TurboMass software of HS-GC-MS’s computer were imported by this way Y data were chosen for chromatogram data and X data was entered for time. PCA for 1 (X) × 12 (Y) matrix consisting of 57,200 data (13 × 4400) were processed in a few seconds.

**Figure 4:**
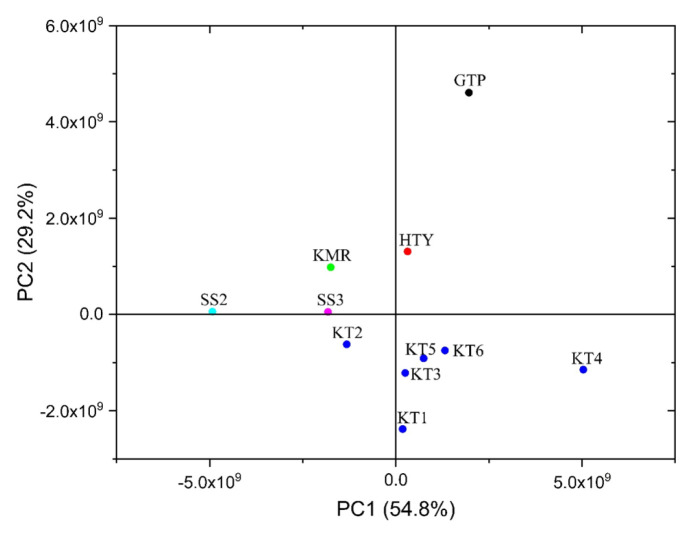
2D score plot of the PCA obtained from chromatogram data of the garlic samples grown in Turkey and two unknown samples (SS2 and SS3).

According to the score plot PCA for chromatogram given in Figure 4, among the Turkish garlic samples, KT mostly extended in the bottom-right quadrant unlike the PCA for relative contents of the VOSCs (see Figure 3). Distribution of the KT samples did not lie in the same quadrants with GTP, HTY, and KMR. Moreover, another remarkable point of the PCA results that the garlics grown in Hatay and Gaziantep, two neighboring cities, were clustered in the upper-right quadrant. On the other hand, unknown samples were clustered in the same region with the sample grown in Kahramanmaraş again like the aforementioned results.

**Figure 5:**
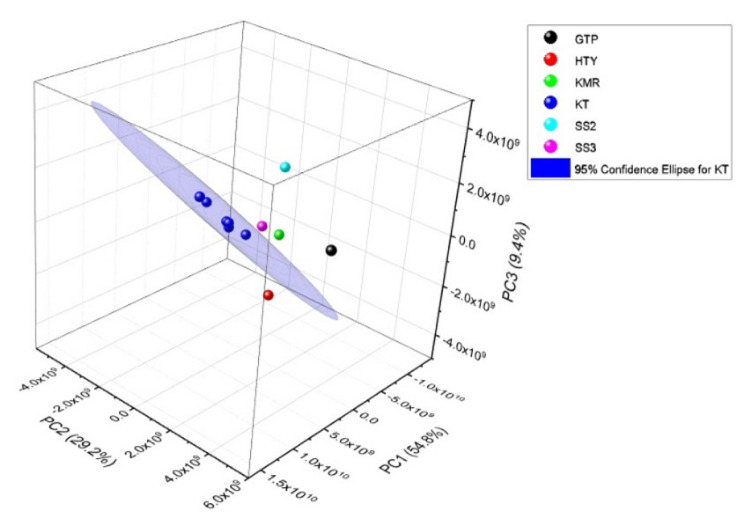
3D PC Analysis result of chromatogram data of the garlic samples grown in Turkey.

As a result of PCA plotted of PC1 versus PC2 explaining 54.8% and 29.2% of the total variance, respectively, five of six KT samples were in the bottom-right quadrant, but only KT2 sample lied in the bottom-left quadrant. Therefore, a 3D PCA was constructed with 93.4% of the cumulative percentage of covariance. According to the 3D PCA performed with chromatographic raw data given in Figure 5, it was concluded that KT samples were completely distinguished from the other Turkish garlic samples.

**Figure 6:**
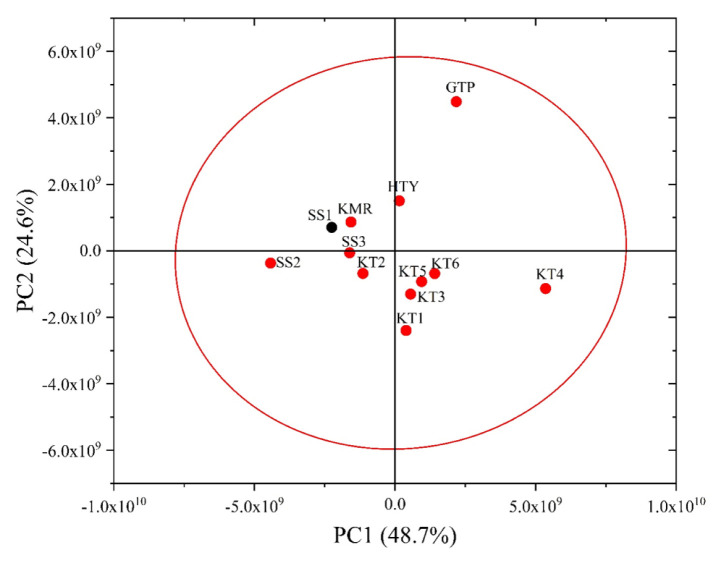
2D PCA score plot constructed with chromatogram raw data of the garlic samples including all SSs.

**Figure 7:**
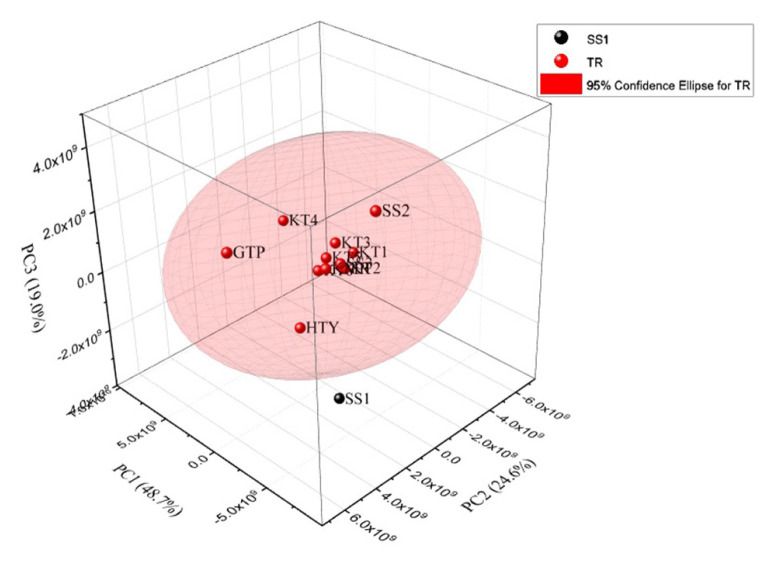
3D PC Analysis result of chromatogram data revealing discrimination of SS1 and the garlic samples grown in Turkey including two unknown samples.

Furthermore, Figure 6 demonstrates 2D PCA where the PC1 and PC2 accounted for 48.7% and 24.6% of the total variance, SS1 was found in 95% confidence level interval with the Turkish samples; that is, SS1 sample could not be separated by this way. However, the cumulative percentage of covariance reached 92.3% by performing 3D PCA (see Figure7) formed with also using the third principal component which explains 19.0% of the total variance. Thus, discrimination of SS1 garlic from Turkish sample was achieved.

## 4. Discussion

The overall results revealed that VOSCs profiling in combination with multivariate analyses are the suitable approaches for the authentication of the geographical origin of garlic. Considering all the results, it can be concluded that the SS1 sample is definitely an imported product, as the seller admits, whereas SS2 and SS3 were Turkish garlic samples and it can even be said that they had been most possibly produced in Kahramanmaraş. It is worth noting that geographical origin separation of garlic samples, especially world-famous “Taşköprü garlic”, were achieved by PCA and HCA techniques applied to HS-GC-MS analysis results of VOSCs. This methodology, thus, does not need any additional examination of VOCs results by utilizing further sample preparation procedures like SPME. In the literature, Mi et al. and Biancolillo et al. classified the garlic samples by using PLS-DA after explorating the data with PCA [13,14]. Both studies utilized HS-SPME-GC-MS systems for analyzing the VOCs. Mi et al. reported that among the 68 of VOCs, 1,2-dimethoxybenzene, 1-(2-methyl-1-cyclopenten-1-yl)-ethanone, mequinol, 2-methoxyphenol, 3,4-dimethylthiophene, 1-allyl-2-(prop-1-en-1-yl)disulfane were assigned as the VOCs that were responsible for separation of the four groups of garlics cultivated in different regions in China [13]. In addition, according to the work presented by Biancolillo et al., though relative (%) contents of some VOSCs were characteristics of several categories, diallyl disulfide and methyl 2-propenyl disulfide (allyl methyl disulfide) were reported as the major compounds among the 13 of VOSCs, and an inverse relationship was found between the content of these two compounds in the garlic classes [14]. Furthermore, relative (%) content of methyl 2-propenyl trisulfide (allyl methyl trisulfide) was the cornerstone for discrimination of KT from the other garlics in this article whereas this compound had been found in the garlics from three different regions in Italy [14]. Like the other studies [13,14], diallyl disulfide was determined as the major compound and its content contributed to discriminating HTY samples from the other samples in Turkey while its content was found significant in two of the four regions in Italy [14]. In addition, di-2-propenyl trisulfide and 1-allyl-2-(prop-1-en-1-yl)disulfane were the determinative compounds especially for the GTP sample whereas the first compound was found in all four regions in Italy [14]. According to Table 3, di-2-propenyl trisulfide was not detected in SS1. Similarly, Mi et al. had not reported any result for di-2-propenyl trisulfide in Chinese garlics [13].

On the other side, it can also be said that the chemometric spectral data analysis performed with the noninvasive ATR-FTIR method is more advantageous than the sophisticated instrumental techniques in terms of ease of sample preparation, low operating cost, and fast operation [17]. In this respect, the proposed study may seem to be disadvantageous, but identification of each volatile compound which is responsible for discriminations can be individually accomplished by gas chromatographic systems. On the other hand, even though HS-GC-MS is an invasive method, this is negligible for garlic samples which are usually easily accessible unless a sample with very low amount sent for examination in forensic or food control laboratories.

As a suggestion, in order to protect the rights of GI registrants, different techniques should be developed for different types of samples apart from the analytical method and the statistical approaches as conducted in this study. Determination of geographical origin by evaluation of a single result obtained from routine analyses is a very difficult issue. Through created databases in the relevant authorities of the states, it may be possible to prepare reports based on objective results with the similar approaches and even using artificial intelligence for a single sample examined in laboratories (forensic, food control, institutes, etc) that act as experts. On the other hand, there is no doubt that the approach proposed in this study can be used to discriminate garlic as well as other species of different genera of the same family from each other. In addition, from the perspective of a forensic chemist, if the sample is a forensic finding, determining its origin may also enable it to be presented as important evidence in a case.

## 5. Conclusion

In the present study, the combinations of HS-GC-MS and chemometrics were investigated for discrimination not only of Taşköprü garlic and the other garlic samples cultivated in different cities in Turkey (Kahramanmaraş, Gaziantep, and Hatay), but also of garlic samples from Turkey and China. To the best of the author’s knowledge, this is the first study to investigate discrimination of geographical origin of well-known Taşköprü garlic by using HCA and PCA to relative content of the VOSCs and to chromatogram raw data. The noteworthy advantages of the methodologies described in this article are as follows: having simple and fast sample preparation procedure, being a cheap and green examination in terms of not applying pretreatments such as liquid-liquid or liquid-solid extractions, rapid statistical analysis of relative contents of VOSCs or directly chromatograms’ raw data consisting of thousands of data, and allowing geographical origin prediction. Consequently, when this study is considered with a holistic approach, the proposed methods have the potential for tracking which can provide reliable supportive results to evaluate the quality and detect frauds of garlic samples.

## Figures and Tables

**Table 1:** The analytical and statistical methods performed in recent studies selected from the literature and in this study.

No	Analysis	Analytical technique	Statistical method	Reference
1	Metabolomics analysis	HPLC-HRMS^1^	PCA^2^, PLS-DA^3^	[12]
2	Multielemental analysis, Volatile compound analysis, metabolomics analysis	ICP-MS^4^, HS-SPME-GC-MS^5^, UHPLC-Q-TOF/MS	PCA, PLS-DA	[13]
3	Organosulfur volatile profiling	HS-SPME-GC-MS	PCA, PLS-DA	[14]
4	Spectral data analysis	HRMAS-NMR^6^,	PCA	[15]
5	Spectral data analysis	HRMAS-NMR	PLS-DA	[16]
6	Spectral data analysis	ATR-FTIR	PLS-DA, SO-CovSel-LDA^7^, SO-PLS-LDA	[17]
7	Elemental profiling	ICP-MS	PCA	[18]
8	Multielemental analysis	ICP-OES^8^	LDA, SIMCA^9^	[19]
9	Trace metal profiling	HR-ICP-MS	SDA^10^	[20]
10	Stable isotopic ratio analysis	EA-IRMS^11^	PCA, PLS-DA	[21,22]
11	Stable isotopic profiling	IRMS	LDA, k-NN^12^	[23]
12	Stable isotopic profiling, multielemental analysis	EA, ICP-MS	DA	[24]
13	Stable isotopic profiling, multielemental analysis	IRMS, ICP-MS	PCA	[25]
14	Stable isotopic profiling, multielemental analysis	EA, EDXRF^13^	DA	[3]
*15*	*VOCs profiling, chromatogram data*	*HS-GC-MS*	*HCA, PCA*	*This Study*

1 High-performance liquid chromatography, high-resolution mass spectrometry,

2 principal component analysis,

3 partial least squares-discriminant analysis,

4 inductively coupled plasma mass spectrometry,

5 Headspace-solid phase microextraction, gas chromatography-mass spectrometry,

6 High-resolution magic angle spinning, nuclear magnetic resonance,

7 Sequential and orthogonalized-covariance selection, linear discriminant analysis,

8 ICP-optical emission spectrometry,

9 Soft independent modeling of class analogy,

10 Stepwise discriminate analysis,

11 Elemental analyzer - isotope ratio mass spectrometer,

12 k-nearest neighbors,

13 Energy dispersive X-ray fluorescence spectrometry

**Table 2:** The operating parameters of the HS-GC-MS system.

Headspace sampler	Headspace pressure	30 psi
	Thermostat Vial temperature	90 °C
	Thermostat time	15 min
	Needle temperature	100 °C
	Transfer Line temperature	110 °C
	Vial Pressurization time	1.00 min
	Injection time	0.06 min
	Withdrawal time	0.20 min
	Sample weight	1.0 g
Gas chromatograph	Injector temperature	220 °C
	Injection type	Split, 10:1
	Carrier gas	Helium, constant flow rate (1 mL/min)
	Oven programme	40 °C for 7 min, 10 °C/min to 220 °C
	Run time	25 min
	Equilibration time	5.0 min
Mass spectrometer	Ionization mode	EI
	EI energy	70 eV
	GC inlet line temperature	200 °C
	Ion source temperature	180 °C
	Function type	Full scan
	Scan range	m/z 20–350
	Solvent delay time	2.5 min

**Table 3:** The relative content (%) of the VOSCs analyzed in the samples by HS-GC-MS and their molecular structures.

		Relative amount (%)											
Compound name	Molecular structure	KT1	KT2	KT3	KT4	KT5	KT6	HTY	GTP	KMR	SS1	SS2	SS3
allyl mercaptan	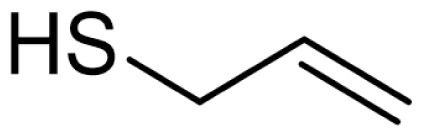	0.21	0.17	0.20	0.15	0.22	0.19	0.24	0.34	0.30	4.69	0.39	0.23
allyl methyl sulfide	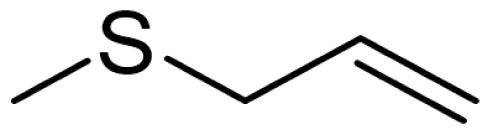	2.22	1.64	2.19	1.50	1.48	1.81	1.82	0.61	1.03	2.59	1.01	0.90
dimethyl disulfide	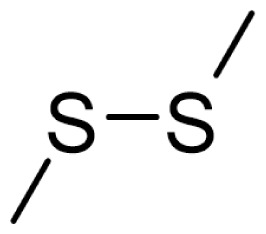	1.69	1.96	2.00	1.41	1.28	1.67	1.36	0.25	0.52	0.57	0.72	0.82
diallyl sulfide	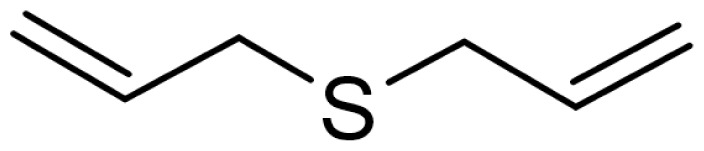	1.49	1.61	1.54	1.49	1.53	1.53	2.04	1.61	1.45	3.47	1.68	1.26
methyl 1-propenyl disulfide	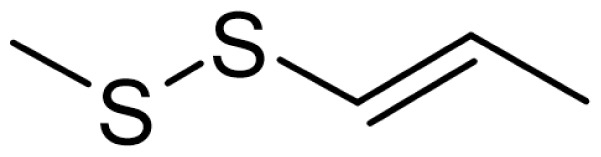	0.36	0.35	0.08	0.25	0.10	0.23	0.30	0.43	0.26	0.00	0.60	0.53
methyl 2-propenyl disulfide	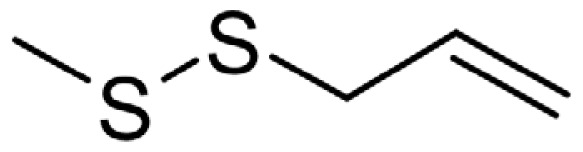	9.36	9.68	9.74	8.02	8.10	8.98	9.19	3.91	6.13	9.38	6.53	6.27
1-methyl-2-(prop-1-en-1-yl)disulfane	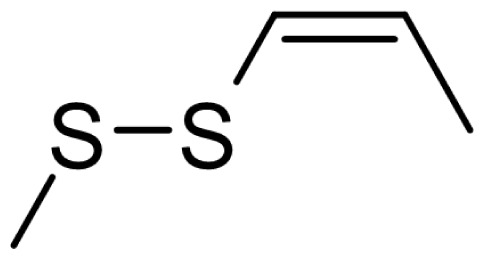	1.23	1.51	0.79	0.96	0.61	1.02	1.49	0.69	1.36	1.03	1.11	0.88
dimethyl trisulfide	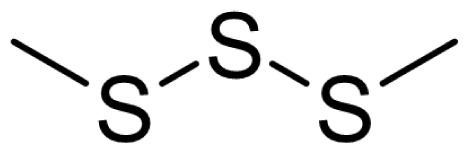	3.11	2.83	3.94	2.64	2.33	2.97	1.98	0.50	0.96	0.00	1.05	1.34
1-allyl-2-isopropyldisulfane	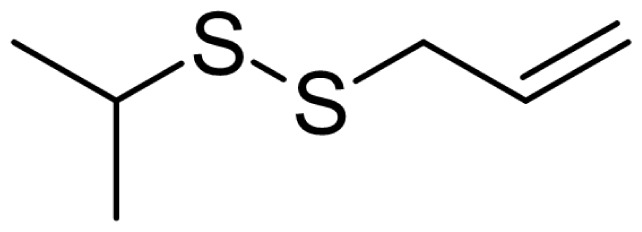	0.06	0.06	0.00	0.03	0.02	0.03	0.02	0.03	0.00	0.18	0.00	0.00
diallyl disulfide	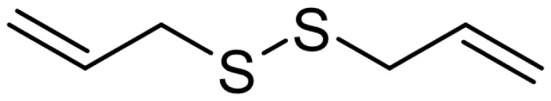	23.72	27.38	23.15	21.22	29.59	25.02	31.93	24.67	31.76	63.44	31.94	29.16
1-allyl-2-(prop-1-en-1-yl)disulfane	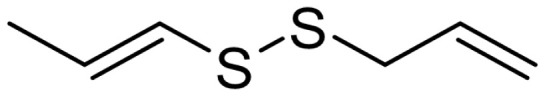	7.13	8.41	3.60	5.81	3.84	5.76	10.80	12.25	13.09	14.17	12.61	14.32
3H-1,2-dithiole	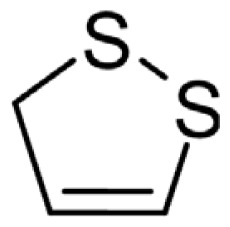	0.49	0.31	0.31	0.43	0.41	0.39	0.31	0.63	0.57	0.00	0.53	0.55
methyl 2-propenyl trisulfide	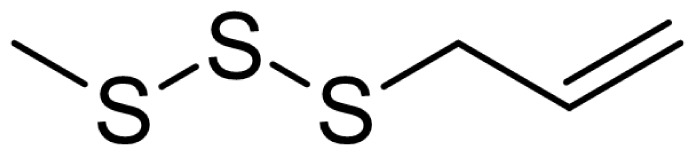	22.06	21.50	24.96	21.43	20.62	22.11	17.24	10.69	13.41	0.48	15.76	16.27
1-allyl-3-propyltrisulfane	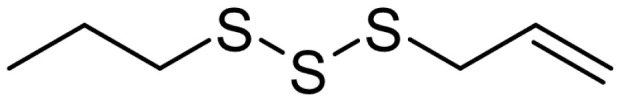	0.10	0.13	0.08	0.10	0.08	0.10	0.06	0.10	0.04	0.00	0.06	0.49
3-vinyl-1,2-dithiacyclohex-4-ene	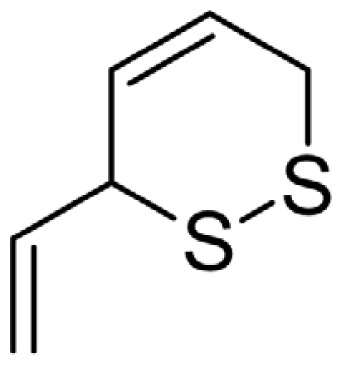	0.82	0.48	0.61	0.47	0.52	0.58	0.41	0.39	0.87	0.00	0.41	1.05
di-2-propenyl trisulfide (alltride)	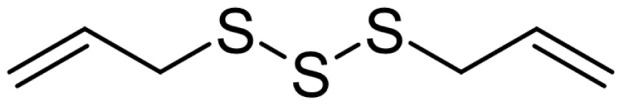	24.45	21.15	25.56	33.09	28.32	26.52	20.03	42.10	26.43	0.00	23.90	23.92
2-vinyl-4H-1,3-dithiine	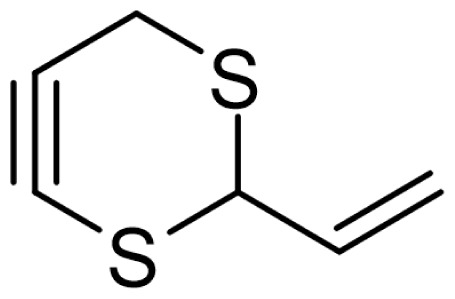	1.50	0.85	1.25	1.01	0.95	1.11	0.77	0.82	1.83	0.00	1.70	2.02
